# Colorful graphene-based wearable e-textiles prepared by co-dyeing cotton fabrics with natural dyes and reduced graphene oxide

**DOI:** 10.1038/s41598-024-52850-6

**Published:** 2024-01-27

**Authors:** Sungwoo Moon, Youngjoo Chae

**Affiliations:** Department of Clothing and Textiles, Chungbuk National University, Chungdae-ro 1, Seowon-gu, Cheongju, Chungbuk 28644 Republic of Korea

**Keywords:** Chemistry, Materials science

## Abstract

In addition to the functionality of electronic textiles (e-textiles), their aesthetic properties should be considered to expand their marketability. In this study, premordanted cotton fabrics were co-dyed with reduced graphene oxide (rGO) and natural dyes to develop ecofriendly and colorful graphene-based wearable e-textiles. The color attributes of the textiles were analyzed in terms of the dyeing conditions, namely, rGO loading, mordant type, and natural dye type. The lightness of the dyed samples increased in the order of cochineal < gardenia blue < rhubarb. Regardless of the natural dye and rGO loading, the lightness of the fabrics mordanted with Fe was lower than that with Al and Cu. Moreover, the rhubarb- and gardenia blue-dyed fabrics exhibited broad chroma and hue dispersions, indicating the strong impact of the dyeing conditions. With increasing rGO loading, the chroma of the rhubarb-dyed fabrics substantially decreased, resulting in decreased color saturation. The initial greenish-blue color of the gardenia blue-dyed fabrics gradually changed to yellowish-green and then yellow. Regardless of the natural dye, drastic overall color changes were observed, with average values of 7.60, 11.14, 12.68, and 13.56 Δ*E*_CMC(2:1)_ at increasing rGO loadings of 1, 3, 5, and 7% owb, respectively.

## Introduction

The progress of the fourth industrial revolution has prompted attempts toward integrating information and communication technology (ICT) into different fields. For example, the global fashion industry is showing considerable interest in the development of smart clothing, which involves the convergence of ICT with fashion. This trend has gained momentum with the rapid expansion of the market for smart and wearable technologies. Smart clothing is designed with embedded sensors and technology that enable it to monitor the surrounding environment, conditions, and stimuli affecting the wearer’s body^1^. These sensors can interact with the environment and the wearer, facilitating a wide range of functions and capabilities. Currently, smart clothing production is being developed for finished products, fabrics, and fibers. The most popular method of manufacturing commercial smart clothing is the physical attachment or embedding of information technology (IT) devices that collect, store, analyze, and transmit data. Although this approach easily imparts smart functions, several drawbacks have been noted in terms of the device weight, wearability, and aesthetic owing to the high stiffness of the device. To address these limitations, researchers have considered the utilization of electronic textiles (e-textiles), in which the fabric itself functions as an IT device, rather than attaching/embedding specialized devices to/into clothing^2–7^.

E-textiles, i.e., textiles with built-in electronic circuits, play an important role in realizing smart clothing by strengthening the human–machine interface through the control of IT devices, virtual/augmented reality, robots, and various electronic products^8^. Because e-textiles are expected to detect signals while maintaining contact with the wearer’s body, their constituent materials should be nontoxic, compatible, and adaptable to diverse external deformations such as human body movements^9^. In addition, e-textiles should have fundamental functions, such as aesthetic appeal, air permeability, ease of washing, and smart functionality. E-textiles are made from electrically conductive materials and can act as devices themselves, eliminating the need for additional devices to achieve electrical functions. Metal-based materials can be added to yarns or fabrics by weaving, knitting, printing, embroidering, and coating and can be utilized as electrically conductive components of e-textiles, thereby achieving a simple fabrication process^10^. However, the development and manufacture of e-textiles face several challenges. In particular, the poor flexibility of metal-based e-textiles makes them unsuitable for use as clothing materials. In addition, electrical conductivity decreases owing to surface oxidation or corrosion when exposed to air and moisture^11^. Researchers have focused on manufacturing e-textiles using carbon-based materials, such as graphene and carbon nanotubes, and nonmetallic materials, such as intrinsically conductive polymers (e.g., polyaniline, polythiophene, poly(3,4-ethylenedioxythiophene) polystyrene sulfonate, and polypyrrole), to overcome the aforementioned limitations^12^.

Graphene is a graphite-derived two-dimensional hexagonal material with the lowest thickness and best electrical, chemical, optical, and mechanical properties among all known materials^13^. The outstanding flexibility, stretchability, and ability to retain electrical conductivity upon physical deformation of graphene demonstrate its applicability as a core material for wearable electronics. Consequently, considerable attention has been focused on the use of graphene as a conductivity booster in e-textiles. Graphene is commonly incorporated into fibers and fabrics by adding it to fiber polymer solutions^14–16^ or depositing it on the surface of fabrics^17–22^. In particular, coating the fabric surface with graphene using traditional dyeing approaches offers the benefits of simplicity and mass production without adversely affecting the elasticity, wearability, and ease of washing of the fabric^22^.

Color is a visual feature that strongly influences human perception and emotions, conveying a considerable amount of information about a given item^23^. In particular, color determines the overall image of clothes, making it one of the factors with the most direct effect on initial customer reactions and purchase choices^24,25^. Similarly, color is used in smart clothing, which prioritizes electrical operation. Regardless of the smart function, smart clothing should have appealing aesthetics, including color, to be marketable. As e-textiles are typical materials for smart clothing, related research and development should focus on external aesthetic features and functional improvement. Graphene-containing fabrics and e-textiles often inherit the black color of graphene, making them less aesthetically appealing. Thus, the color attributes and color diversity of graphene-containing fabrics should be thoroughly examined. However, to date, limited studies have been conducted on the color attributes of graphene-containing fabrics.

Shateri-Khalilabad and Yazdanshenas^19^ analyzed the physical color attributes of a graphene oxide (GO)-coated cotton fabric, which was dyed by dipping into an aqueous GO dispersion, and graphene-coated cotton fabric, which was prepared by the immersion of the GO-coated fabric into an aqueous solution of reducing agents. Both GO- and graphene-coated samples were darker than the untreated fabric based on their lower lightness (L*) values. In particular, the GO-coated fabric was more reddish and yellowish than the untreated fabric, primarily because of the elevated redness–greenness (a*) and yellowness–blueness (b*) values, respectively. Fan et al*.*^26^ measured the L*, a*, b*, chroma (C*), and color strength (*K/S*) values as the color attributes of a polyester fabric dyed by reducing dyeing solutions, which were obtained by mixing a disperse blue dye with GO at loadings of 0.5, 1, 2, 3, and 4% on mass fiber. With increasing loading of reduced graphene oxide (rGO), the L* and a* values of the blue fabric increased, indicating an increase in the lightness and redness, whereas the *K/S* value decreased. These previous studies quantitatively investigated the effects of the graphene type and loading for dyeing on the color attributes of graphene-based e-textiles, which are yet to be completely explored. However, in most of the studies, only achromatic graphene, GO, and rGO have been employed, and multiple colorful dyes are yet to be utilized. Consequently, related findings provide limited insights on the analysis of color properties and design of colorful e-textiles.

In addition to textile dyeing, Luo et al.^27^ experimented graphene-based hair dyeing, revealing that GO- and rGO-based dyes produce different shades of brown and black colors, while providing antistatic performance, improved thermal conduction, and colorfastness. As hair can be classified as a fiber, these results exhibit that graphene dyeing into textiles can obtain e-textiles with diverse functions and colors that affect the overall comfort of the wearer and electrical conductivity of the material. rGO is considered suitable for use in clothing products that are often washed because of its excellent washing resistance in terms of color and antistatic performance^27^. Furthermore, the gradual color change from transparent to black upon GO and rGO reduction allows precise gradation dyeing based on the reduction degree control^27^.

Despite their safety toward humans and the environment, natural dyes and graphene have not yet been combined to obtain various colors. In particular, although various synthetic dyes have been developed, natural dyes are still used in the textile industry due to their sustainability and elegant colors, and thus the related studies have been continuously conducted including those on the isolation method of colorants from natural resources such as plants^28–36^. In this study, cotton fabrics were co-dyed with rGO and three natural dyes under various conditions to develop ecofriendly and colorful graphene-based wearable e-textiles. Subsequently, their physical color attributes, namely, lightness, chroma, hue, and overall color, were analyzed as functions of the dyeing parameters, namely, dye type, mordant type, and rGO loading. Also, the colorfastness to laundering of the dyed cotton fabrics was evaluated.

## Methods

### Materials

Desized, scoured, and bleached plain-woven fabrics (100% cotton; Silkville Co., Ltd., Republic of Korea) with a yarn density of 96 (warp) × 96 (weft)/in^2^ and size of 5 cm × 5 cm were used as substrates for co-dyeing. Cochineal (red), rhubarb (yellow), and gardenia blue (blue) powders were used as natural dyes, and KAl(SO_4_)_2_·12H_2_O, CuSO_4_·5H_2_O, and FeSO_4_·*x*H_2_O were used as the mordants. The reason why the metallic mordants were used in this study is that they can effectively bind natural dyes to fibers at a low cost and in a relatively simple manner. Repon et al.^37^ stated that economical and highly efficient natural dyeing is possible with the minimal adverse effects of metallic mordants on the environment and human health when they are used at a safe concentration during dyeing. The natural dyes and mordants were purchased from Oldbrown Co., Ltd. (Republic of Korea) and Silkville Co., Ltd. (Republic of Korea), respectively. The rGO dispersion (0.5 wt%) used to prepare the dyeing solutions was purchased from Standard Graphene Inc. (South Korea).

### Co-dyeing with natural dyes and rGO

**Figure 1:**
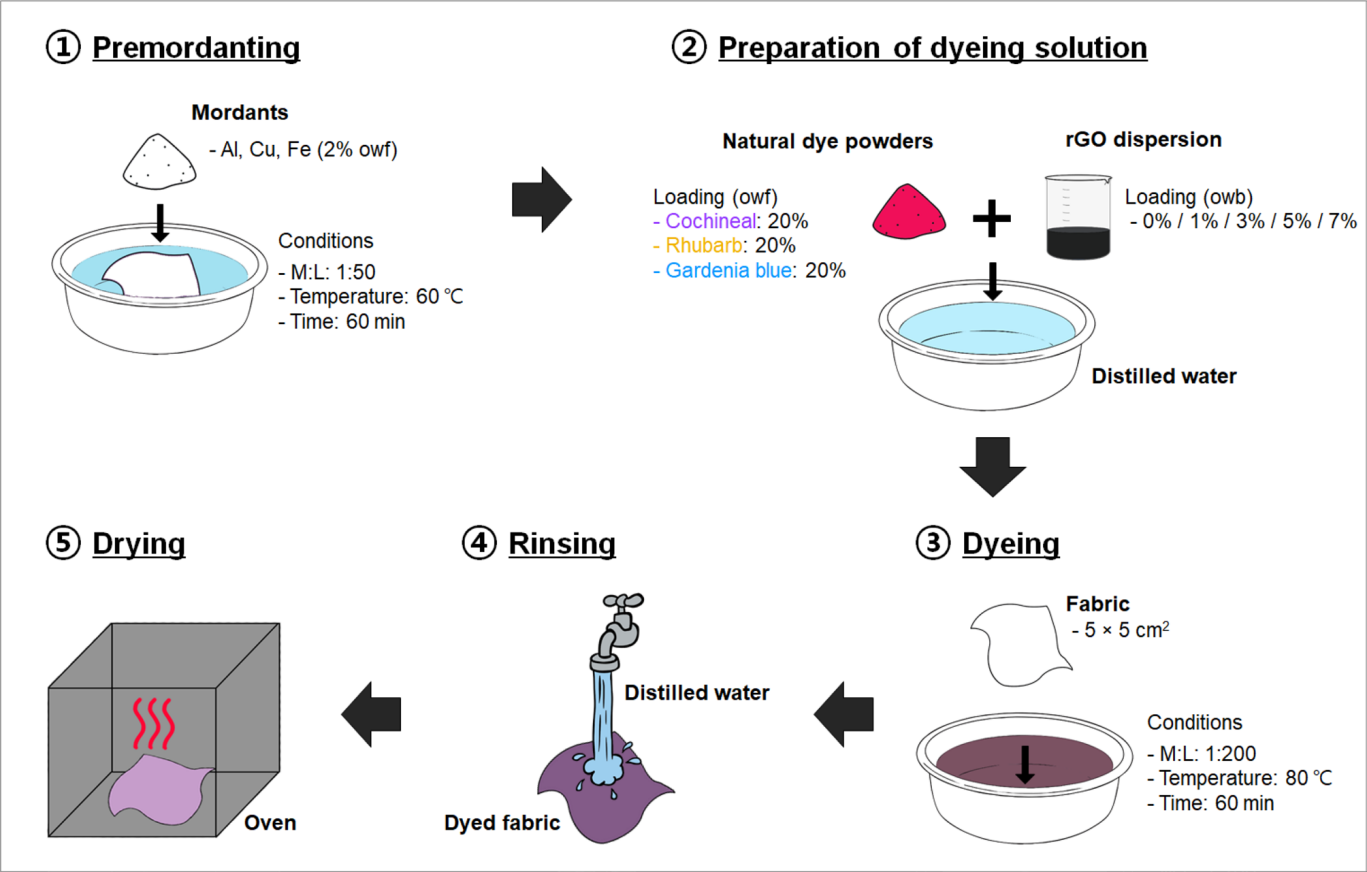
Schematic of the dyeing process.

**Figure 2:**
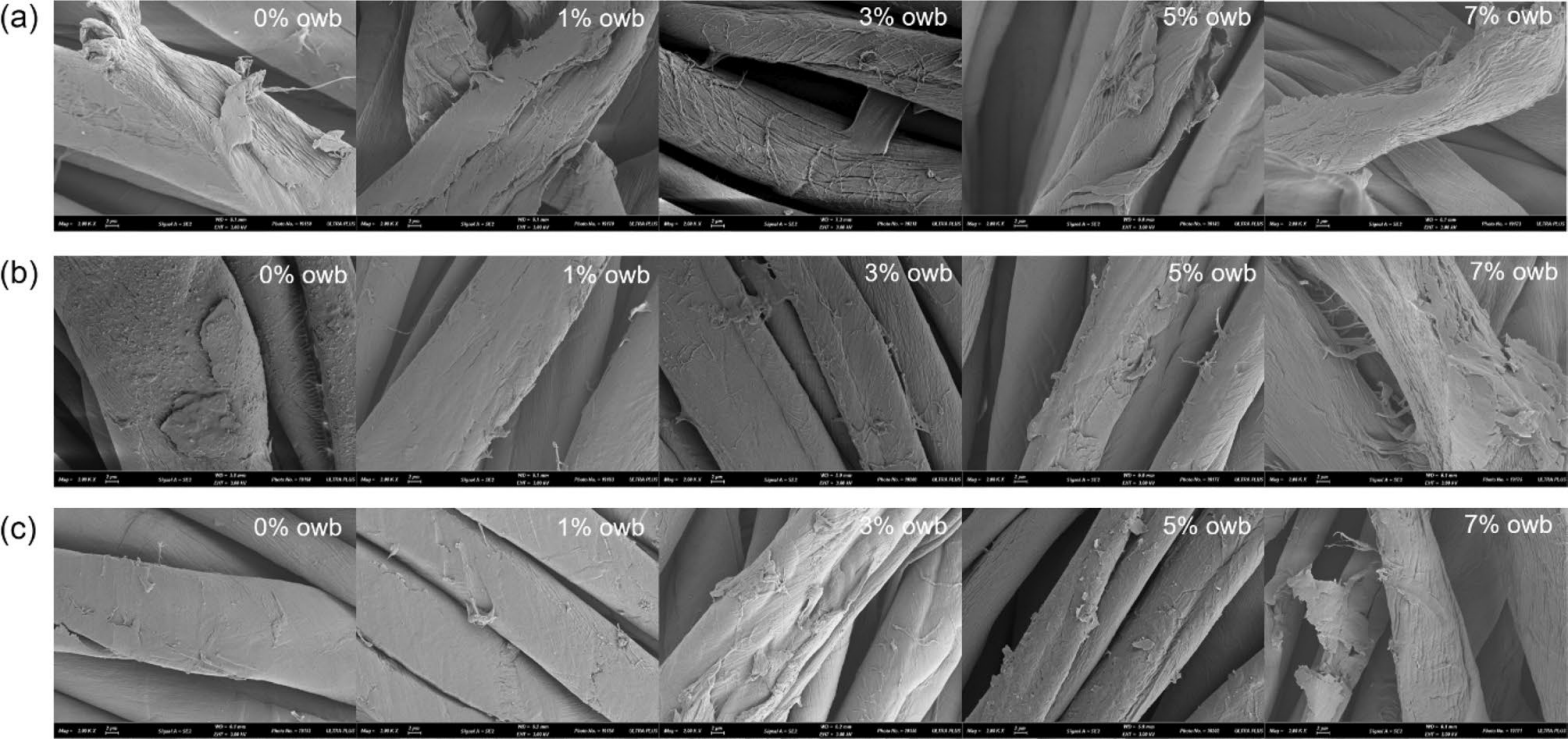
SEM images of the fabrics co-dyed with rGO and natural dyes: **(a)** cochineal-dyed fabrics, **(b)** rhubarb-dyed fabrics, and **(c)** gardenia blue-dyed fabrics. The rGO loading (% owb) increases from left (0% owb) to right (7% owb).

After premordanting at a bath ratio of 1:50 and temperature of 60 °C for 60 min using 2% on the weight of fabric (owf) mordant dispersions, the fabrics were naturally dried at room temperature and then dyed. The dyeing process was initiated by heating distilled water. Subsequently, one of the natural dyes (cochineal, rhubarb, or gardenia blue) and rGO dispersion of different amounts were mixed with the heated distilled water. Dyeing was carried out at 80 °C for 60 min at a bath ratio of 1:200 and dye loading of 20% owf. The bath ratio of 1:200 was the optimal ratio determined through several rounds of preliminary experiments to obtain both aesthetic properties and electrical performance. The loading of the rGO dispersion in the dyeing solution was set to 1%, 3%, 5%, and 7% on the weight of bath (owb). In addition, a control sample was obtained by dyeing without rGO (0% owb). The dyed fabrics were washed with distilled water and oven-dried overnight at 60 °C. A total of 45 dyed fabrics (3 natural dyes × 3 mordants × 5 rGO loadings) was obtained. Figure 1 illustrates the dyeing procedure employed in this study, and Fig. 2 shows the scanning electron microscopy (SEM) images of the dyed fabrics. As can be seen in Fig. 2, the introduction of rGO shifted the surface of the dyed fibers.

### Measurement and analysis of physical color

The physical color attributes of the dyed cotton fabrics were measured using a Konica Minolta CM-26d spectrophotometer (Japan). The sample reflectance was measured at intervals of 10 nm in the visible-light region (360–740 nm) under the conditions of a large aperture (MAV: 8 mm), specular component included (SCI), and ultraviolet exclusion. When the measurement was conducted, five different areas on the surface of each dyed fabric were measured, and then the measured values were compared to evaluate the color quality of the dyed fabric. As a result, the average difference between the measured colors of the five areas was 3.65 ΔE*_ab_, which was lower than the color discrimination threshold of human vision for textiles previously reported by Chae & Moon^38^. This indicates that different colors of the dyed fabrics were generally obtained uniformly under different dyeing conditions. The obtained reflectance values were converted to *Commission Internationale de l'Eclairage* (CIE) values of L*_10_, a*_10_, b*_10_, C*_ab,10_, and *h*_ab,10_ based on the CIE 10° standard observer and CIE standard illuminant D65.

The physical color attributes of the dyed fabrics were compared and analyzed according to the type of the natural dye (cochineal, rhubarb, and gardenia blue), mordant (Al, Cu, and Fe), and rGO loading (0%, 1%, 3%, 5%, and 7% owb). For each mordant, the color differences, i.e., ΔL*_10_, Δa^*^_10_, Δb^*^_10_, ΔC^*^_ab,10_, and Δ*h*_ab,10_, between the fabrics dyed using natural dyes with and without rGO were calculated using Eqs. (1)–(5) and used to determine Δ*E*_CMC(2:1)_ (Eqs. (6)–(12)).

1 $$ \Delta L_{10 }^{*} = L_{10,Bat}^{*} - L_{10, Std }^{*} $$

2 $$ \Delta a_{10 }^{*} = a_{10,Bat }^{*} - a_{10, Std }^{*} $$

3 $$ \Delta b_{10 }^{*} = b_{10,Bat }^{*} - b_{10, Std}^{*} $$

4 $$ \Delta C_{ab,10}^{*} = C_{ab,10, Bat }^{*} - C_{ab,10, Std}^{*} $$

5 $$ \Delta h_{ab,10} = h_{ab,10,Bat} - h_{ab,10,Std} $$

6 $$ \Delta E_{{CMC\left( {2:1} \right)}} = \sqrt {\left( {\frac{{\Delta L_{10 }^{*} }}{{2S_{L} }}} \right)^{2 } + \left( {\frac{{\Delta C_{ab,10 }^{*} }}{{S_{C} }}} \right)^{2 } + \left( {\frac{{\Delta H_{ab,10}^{*} }}{{S_{H} }}} \right)^{2} } $$

where

7 $$ \Delta H_{ab,10}^{*} = \left( {\frac{\begin{gathered} \sqrt[2]{{C_{ab,10, Bat}^{*} C_{ab,10, Std}^{*} }} \cdot { }\sin \left( {\frac{\pi }{180} \cdot \frac{{\Delta h_{ab,10} }}{2}} \right) \hfill \\ or \hfill \\ \left( {a_{10,Bat}^{*} b_{10, Std}^{*} - a_{10, Std}^{*} b_{10,Bat}^{*} } \right) \hfill \\ \end{gathered} }{{\left[ {0.5\left( {C_{ab,10, Bat}^{*} C_{ab,10, Std}^{*} + a_{10,Bat}^{*} a_{10, Std}^{*} + b_{10, Std}^{*} b_{10,Bat}^{*} } \right)} \right]^{1/2} }}} \right) $$

8 $$ S_{L} = \left\{ {\begin{array}{*{20}l} {\frac{{0.040975L_{10,Std}^{*} }}{{1{ } + { }0.01765L_{10,Std}^{*} }}} \hfill & {L_{10,Std}^{*} \ge 16} \hfill \\ {0.511} \hfill & {L_{10,Std}^{*} < 16} \hfill \\ \end{array} } \right\} $$

9 $$ S_{C} = \frac{{0.0638 C_{ab,10,Std}^{*} }}{{1 + 0.0131 C_{ab,10,Std}^{*} }}+0.638 $$

10 $$ S_{H} = S_{C} \left( {Tf + { 1 } - f} \right) $$

11 $$ f = \sqrt {\frac{{\left( {C_{ab,10,Std}^{*} } \right)^{4} }}{{\left( {C_{ab,10,Std}^{*} } \right)^{4} + 1900}}} $$

12 $$ T = \left\{ {\begin{array}{*{20}c} {0.56 + \left| {0.2\cos \left( {h_{{ab,10,{\text{Std}}}} + 168^\circ } \right)} \right| 164^\circ \le h_{{ab,10,{\text{Std}}}} \le 345^\circ } \\ {0.36 + \left| {0.4\cos \left( {h_{{ab,10,{\text{Std}}}} + 35^\circ } \right)} \right|{\text{ otherwise }}} \\ \end{array} } \right\} $$

where subscripts “Bat” and “Std” refer to the fabrics dyed with and without rGO, respectively.

Statistical analysis was performed to examine the effects of co-dyeing on the color changes using Pearson’s correlation analysis. The significant effects revealed by the correlation analysis were further quantified using simple regression analysis and one-way ANOVA with Duncan’s post-hoc test.

### Evaluation of colorfastness

The colorfastness of the dyed cotton fabrics was evaluated based on the test method for colorfastness to laundering suggested in *American Association of Textile Chemists and Colorists* (AATCC) 61:2020^39^. The 45 dyed fabrics with a size of 5 cm × 5 cm were agitated with 50 stainless steel balls in a Samsung WW90T3000KW Laundering machine (Republic of Korea) at 50 °C for 45 min at a liquor detergent ratio of 0.23% relative to the volume of water with resulting color staining. The color staining was graded on a scale of 1 to 5 using AATCC Grayscale for Color Staining, with 5 indicating the highest quality and 1, the lowest. A rating of 3 or higher for color staining is considered acceptable by the *American Society for Testing of Materials* (ASTM) for apparel and home goods.

## Results and discussion

### Physical color attributes of co-dyed fabrics

**Figure 3:**
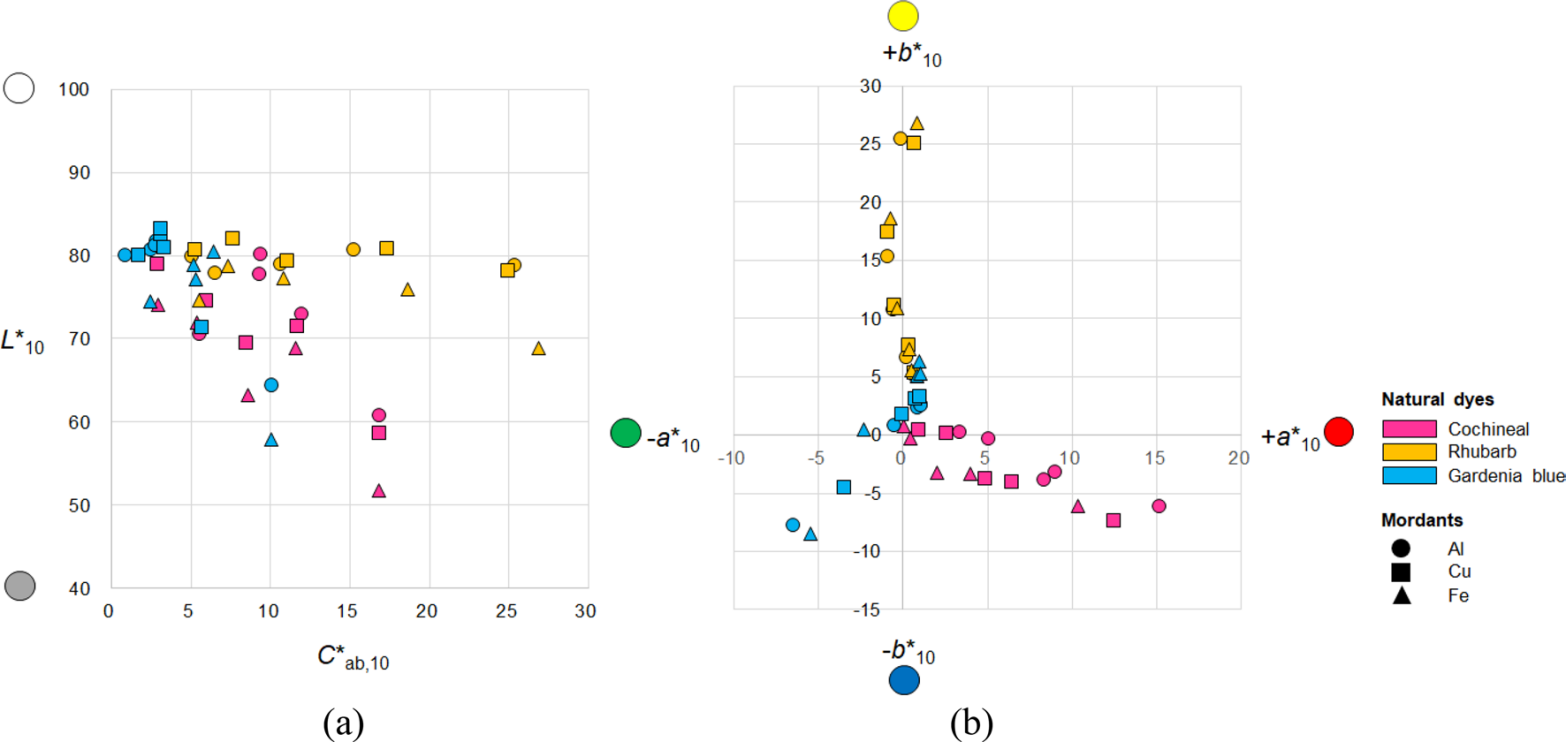
Distribution of 45 dyed cotton fabrics in the CIELAB color space: (a) L*_10_–C*_ab,10_ and (b) a*_10_–b*_10_ planes.

Tables 1, 2 and 3 present the images and physical color attributes of the dyed fabrics. Figure 3 shows the distribution of the fabrics in the CIELAB color space.

**Table 1:**
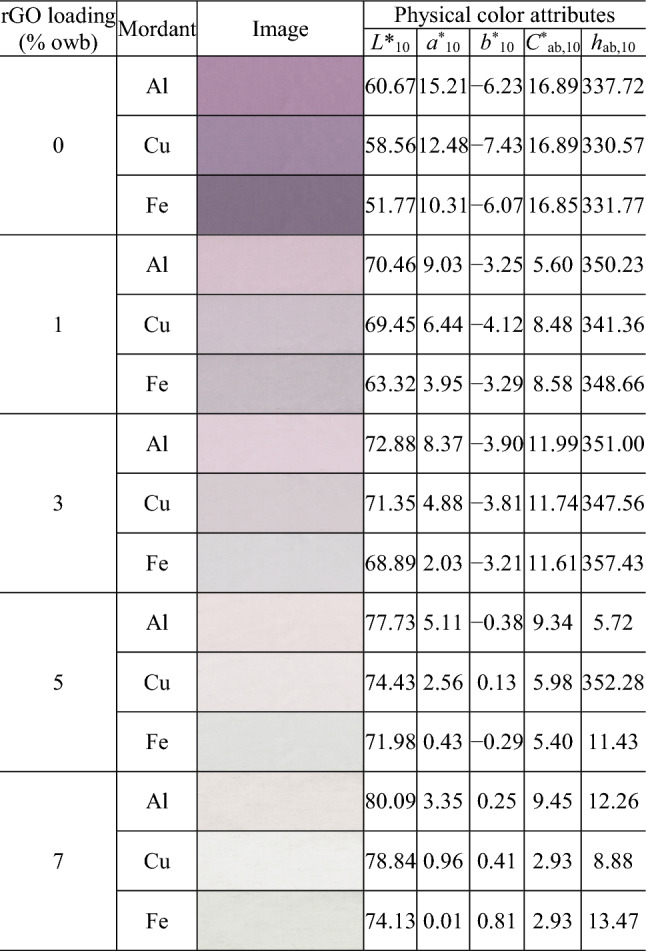
Physical color attributes of the cochineal-dyed fabrics.

**Table 2:**
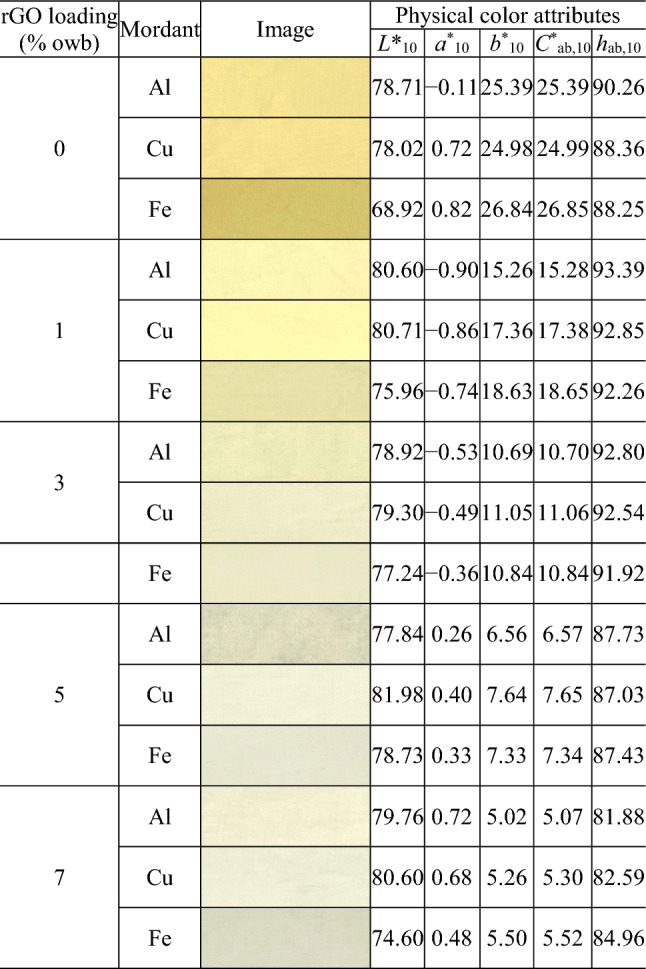
Physical color attributes of the rhubarb-dyed fabrics.

**Table 3:**
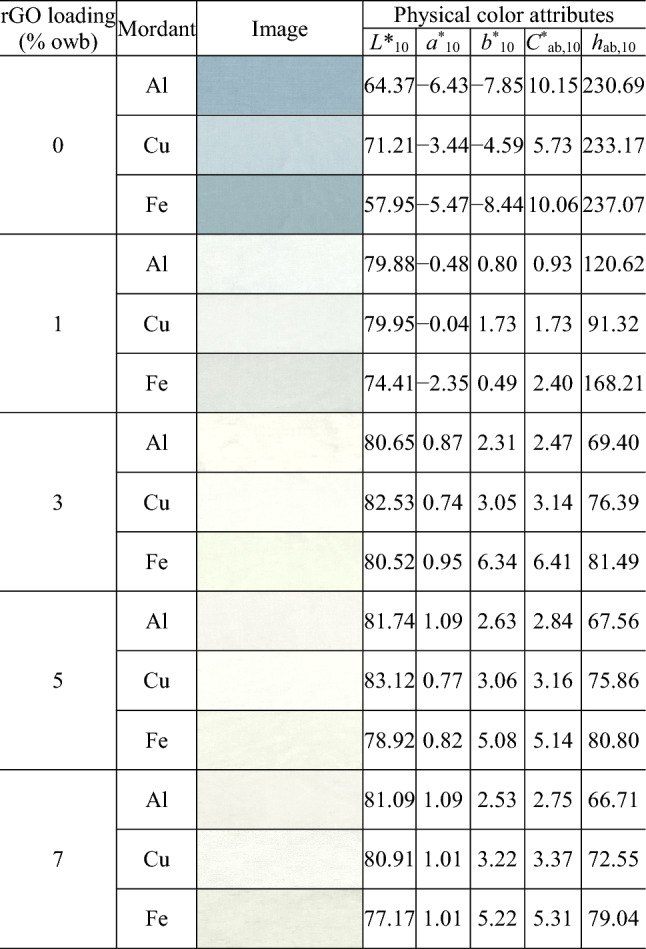
Physical color attributes of the gardenia blue-dyed fabrics.

As shown in Fig. 3, the dyed cotton fabrics had a wide distribution of lightness (Fig. 3a), chroma (Fig. 3a), and hue (Fig. 3b; hue (*h*_ab,10_) was calculated from redness–greenness (a^*^_10_) and yellowness–blueness (b^*^_10_)). The cochineal-, rhubarb-, and gardenia blue-dyed fabrics had the L^*^_10_ values of 51.77–80.09 (average = 69.64; standard deviation (SD) = 7.95), 68.92–81.98 (average = 78.13; SD = 3.18), and 57.95–83.12 (average = 76.96; SD = 7.23), respectively. Therefore, the rhubarb-dyed fabrics were the lightest, whereas the cochineal-dyed fabrics were the darkest. Lightness also depends on the type of mordants. In particular, regardless of the type of natural dyes and rGO loading, the lightness (L^*^_10_) values of the Fe-mordanted fabrics were lower than those of the Al- and Cu-mordanted fabrics (by 4.91 on an average; L^*^_10,Al-mordanted fabrics_ − L^*^_10,Fe-mordanted fabrics_ =  − 4.72; L^*^_10,Cu-mordanted fabrics_ − L^*^_10,Fe-mordanted fabrics_ =  − 5.10), i.e., the Fe-mordanted fabrics were darker.

Rhubarb- and gardenia blue-dyed fabrics had the widest and narrowest chroma (C^*^_ab,10_) ranges of 21.77 (5.07–26.85) and 9.22 (0.93–10.15), respectively, i.e., the most and least affected chroma based on the type of mordants and rGO loading used during dyeing, respectively. As shown in Fig. 3b, the dyed fabrics are positioned in different hue quadrants [first quadrant (+ a^*^_10_, + b^*^_10_): red-yellow; second quadrant (− a^*^_10_, + b^*^_10_): yellow-green; third quadrant (− a^*^_10_, − b^*^_10_): green–blue; and fourth quadrant (+ a^*^_10_, − b^*^_10_): blue-red]. The cochineal-dyed fabrics are located in the first and fourth quadrants, i.e., they are either yellowish red (most yellowish red values: a^*^_10_ = 0.01, b^*^_10_ = 0.81, *h*_ab,10_ = 13.47) or bluish red (most bluish red values: a^*^_10_ = 12.48, b^*^_10_ =  − 7.43, *h*_ab,10_ = 330.57). In contrast, the rhubarb-dyed fabrics are located in the first and second quadrants, i.e., they are either reddish yellow (most reddish yellow values: a^*^_10_ = 0.72, b^*^_10_ = 5.02, *h*_ab,10_ = 81.88) or greenish yellow (most greenish yellow values: a^*^_10_ =  − 0.90, b^*^_10_ = 15.26, *h*_ab,10_ = 93.39). Unlike the cochineal- and rhubarb-dyed fabrics, the gardenia blue-dyed fabrics are widely distributed across three (first, second, and third) quadrants, i.e., they are yellowish, greenish, or green–blue, thereby covering a broader hue range. This indicates that when co-dyed with rGO, the gardenia blue dye can create textiles with more diverse hues by changing the dyeing conditions than the other natural dyes.

### Factors influencing the changes in physical color attributes of co-dyed fabrics

Pearson’s correlation analysis was performed using the rGO loading and mordant type as independent variables, whereas color differences between the fabrics dyed with and without rGO [ΔL*_10_, ΔC^*^_ab,10_, Δ*h*_ab,10_, and overall color difference (Δ*E*_CMC(2:1)_)] were considered as dependent variables. The absolute values of ΔL*_10_, ΔC^*^_ab,10_, and Δ*h*_ab,10_ were used in the analysis to depict the magnitude of changes, not their direction. The original values of Δ*E*_CMC(2:1)_ were used because this parameter is positive by definition.

Table 4 presents the Pearson correlation coefficients for the studied variables. The properties of the dyed cotton fabrics were found to be affected by the rGO loading and mordant type. The L*_10_, *h*_ab,10_, and *E*_CMC(2:1)_ of the cochineal-dyed fabrics were significantly affected by rGO loading. Meanwhile, for the rhubarb-dyed fabrics, rGO loading significantly affects the C^*^_ab,10_ and *E*_CMC(2:1)_ values, whereas the mordant type significantly influences the L*_10_ values. Finally, for the gardenia blue-dyed fabrics, only rGO loading has a significant effect on *h*_ab,10_. Thus, the mordant type has a significant effect on the L*_10_ values of the rhubarb-dyed fabrics.

**Table 4:** Pearson’s correlation coefficients for the studied variables.

Dependent variables	Independent variables	
	rGO loading (% owb)	Mordant
$$\left| {\Delta L^{*}_{10} } \right|$$		
Cochineal-dyed	0.919**	0.320
Rhubarb-dyed	− 0.025	0.895**
Gardenia blue-dyed	0.133	0.326
Total	0.208	0.266
$$\left| {\Delta C^{*}_{ab, 10} } \right|$$		
Cochineal-dyed	0.442	0.254
Rhubarb-dyed	0.952**	0.025
Gardenia blue-dyed	− 0.296	− 0.472
Total	0.308	− 0.011
$$\left| {\Delta h_{ab, 10} } \right|$$		
Cochineal-dyed	0.883**	0.335
Rhubarb-dyed	0.226	− 0.254
Gardenia blue-dyed	0.668*	− 0.224
Total	0.145	− 0.015
Δ*E*_CMC(2:1)_		
Cochineal-dyed	0.922**	0.349
Rhubarb-dyed	0.949**	0.081
Gardenia blue-dyed	0.387	0.398
Total	0.507**	0.228

“Total” represents the overall trend for all natural dyes.

*P < 0.05; **P < 0.01.

### Effects of rGO loading and mordant type on the color changes of co-dyed fabrics

A simple regression analysis on the effects of rGO loading and one-way analysis of variance (ANOVA) with Duncan’s post-hoc test on the effects of mordant type were performed to further examine the trends revealed by Pearson’s correlation analysis. Unlike the correlation analysis, the relative values of ΔL*_10_ and ΔC^*^_ab,10_ were used to consider the direction of significant effects. Moreover, both relative and absolute values of Δ*h*_ab,10_ were used because *h*_ab,10_ reflects multiple color attributes, that is, the magnitudes of red (*h*_ab,10_ = 0 or 360), yellow (*h*_ab,10_ = 90), green (*h*_ab,10_ = 180), and blue (*h*_ab,10_ = 270), which are arranged orthogonally in the hue circle to afford four quadrants^40^. Therefore, it may be meaningless to discuss whether Δ*h*_ab,10_ is positive or negative based on the difference between the hues depending on their quadrants.

#### Lightness changes

**Figure 4:**
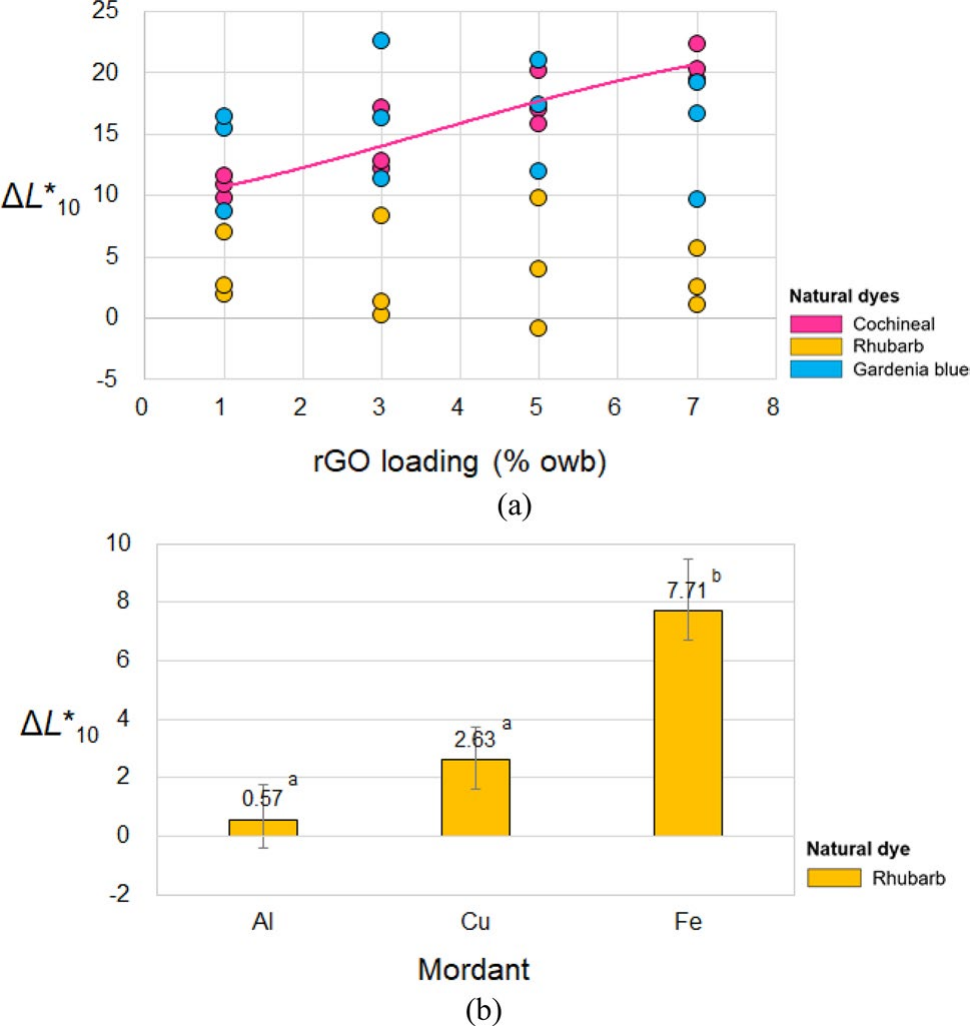
Effects of (**a**) rGO loading and (**b**) mordant type on the ΔL*_10_ of the dyed fabrics. The solid line in (**a**) shows the best fit for the cochineal-dyed fabric, which has the most significant effect.

Figure 4 presents the effects of rGO loading and mordant type on ΔL*_10_. According to Table 4, the color attributes of the cochineal-dyed fabrics are more strongly influenced by rGO loading than those of the other two types of naturally dyed fabrics. The L*_10_ of the cochineal-dyed fabrics increases with the rGO loading (Fig. 4a). Although no statistically significant effects are observed for the other two natural dyes (Tables 2, 3), there are more cases with a positive correlation between the rGO loading and lightness than those with a negative correlation. This finding contradicts previous studies^19,20^, in which fabric dyeing was performed only with graphene without colorful dyes. In these studies, L*_10_ decreased upon dyeing with GO and graphene with increasing loading amount. In contrast, Fan et al.^26^ reported an increase in L*_10_ with an increase in rGO loading for a fabric co-dyed with a disperse blue dye and rGO. Based on these results, dyeing with graphene results in a darker fabric because of the black color of graphene, whereas the use of graphene in combination with a colorful dye increases the lightness of the fabric because of the interaction between the two pigments.

The mordant type has a significant effect on ΔL*_10_ only for the rhubarb-dyed fabrics. As shown in Figure 4b, the fabrics co-dyed with rhubarb and rGO have positive ΔL*_10_ values, regardless of the mordant type, indicating that they are lighter than the fabrics dyed with rhubarb without rGO. In particular, the highest increase in ΔL*_10_ was observed for the Fe mordant, in which ΔL*_10_ was 7.71 (S.D.: 1.76). The post-hoc test performed for the Al- and Cu-mordanted fabrics revealed that there is no statistically significant difference in the increase in ΔL*_10_, which is equal to the average value of 1.6 [Al-mordanted fabric: 0.57 (S.D.: 1.18); Cu-mordanted fabric: 2.63 (S.D.: 1.09)].

#### Chroma changes

**Figure 5:**
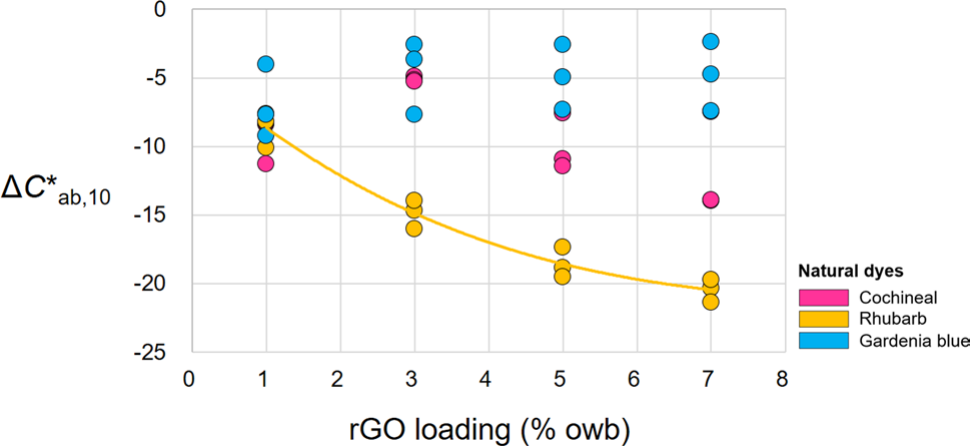
Effect of rGO loading on the ΔC^*^_ab,10_ of the dyed fabrics. The solid line shows the best fit for the rhubarb-dyed fabric, which has the most significant effect.

Figure 5 presents the effect of rGO loading on the ΔC^*^_ab,10_ of the dyed fabrics. Negative ΔC^*^_ab,10_ values were observed for all naturally dyed fabrics. The chroma of the fabrics dyed without rGO exceeded that with rGO. Among the three naturally dyed fabrics, only the rhubarb-dyed fabrics demonstrated a significant effect of rGO loading on ΔC^*^_ab,10_. In particular, the decrease in C^*^_ab,10_ due to dyeing with a mixture of rhubarb and rGO became more pronounced at higher rGO loadings, with the average ΔC^*^_ab,10_ of −8.64 (S.D.: 1.30) and −20.44 (S.D.: 0.83) at rGO loadings of 1% and 7%, respectively. This trend is consistent with the results of a previous study^26^, in which a fabric co-dyed using a disperse blue dye and rGO exhibited C^*^_ab,10_ values of 18.29, 15.89, and 15.55 at rGO loadings of 0.5%, 2.0%, and 4.0%, respectively. However, the effect of rGO loading on ΔC^*^_ab,10_ was found to be less pronounced than that observed in our study.

#### Hue changes

**Figure 6:**
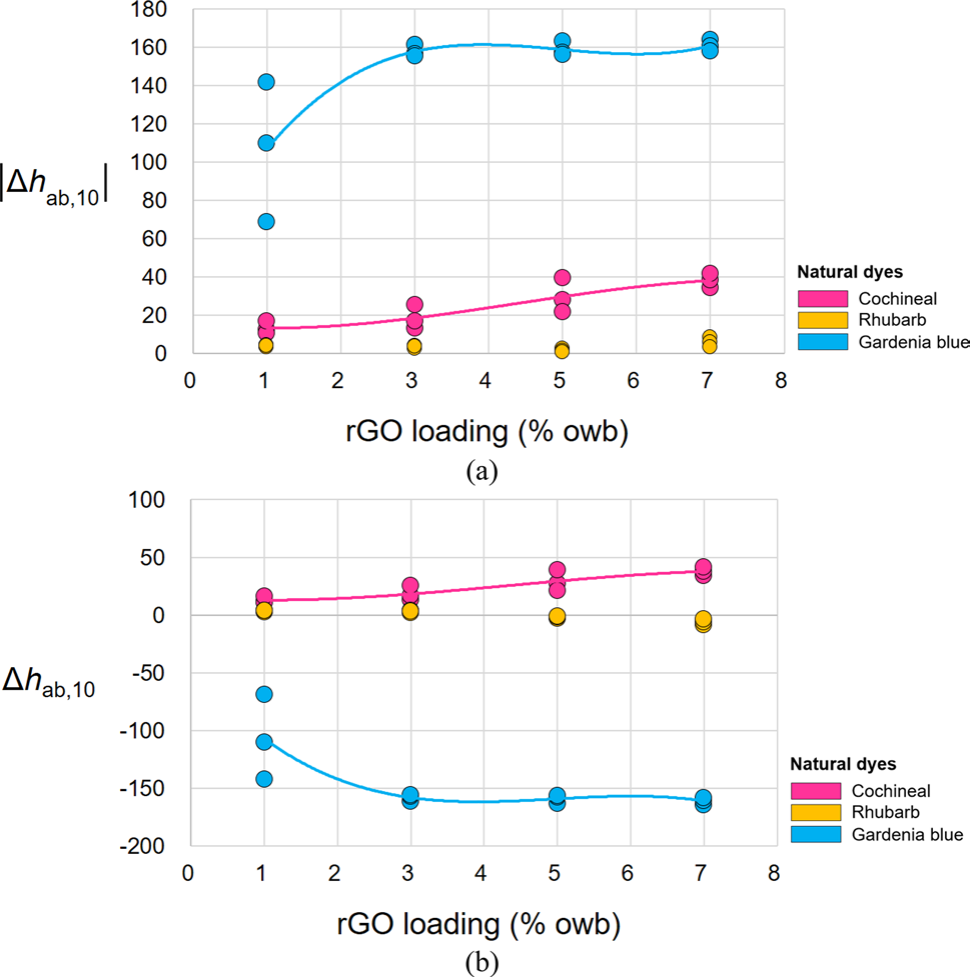
Effect of rGO loading on the (**a**)$\left| {\Delta h_{ab,10 } } \right|$ and (**b**) Δ*h*_ab,10_ of the dyed fabrics. The solid lines show the best fits for the cochineal- and gardenia blue-dyed fabrics, which had significant effects.

Figure 6 shows the effects of rGO loading on the Δ*h*_ab,10_ of the dyed fabrics, whereby statistically significant effects are noted for the cochineal- and gardenia blue-dyed fabrics. Figure 6a, b show the magnitude and direction of Δ*h*_ab,10_, respectively. Although the introduction of rGO deteriorated the C^*^_ab,10_ of the rhubarb-dyed fabric, it induced negligible effects on its original yellowish hue (*h*_ab,10_ ≈ 90, Table 2). The magnitude of Δ*h*_ab,10_ increased with increasing rGO loading for the cochineal- and gardenia blue-dyed fabrics (Fig. 4a). For the cochineal-dyed fabric, $\left| {\Delta h_{ab,10 } } \right|$ was 13.40 (S.D.: 3.15) and 38.18 (S.D.: 3.58) at rGO loadings of 1% and 7%, respectively, depicting the increase with the rGO loading. For the gardenia blue-dyed fabric, $\left| {\Delta h_{ab,10 } } \right|$ sharply increased from 106.93 (S.D.: 36.60) to 157.88 (S.D.: 3.01) as the rGO loading was increased from 1 to 3%, respectively. However, a further increase in rGO loading had no significant effects $[\left| {\Delta h_{ab,10 } } \right|$= 158.90 (S.D.: 3.70) and 160.88 (S.D.: 2.98) at rGO loadings of 5% and 7%, respectively]. Thus, the average Δ*h*_ab,10_ was significantly higher for the gardenia blue-dyed fabrics than that for the cochineal-dyed fabrics. In the former, the addition of rGO shifted *h*_ab,10_ across two quadrants of the *h*_ab,10_ circle.

The initial *h*_ab,10_ of the fabric dyed with cochineal without rGO was blue-red (*h*_ab,10_ = 330.57–337.72, Table 1). Increasing rGO loading shifted the hue of the dyed fabric to primary red. Moreover, the fabric dyed with gardenia blue without rGO initially had a green-bluish hue (*h*_ab,10_ = 230.69–237.07; Table 3). When the rGO loading was increased to 3%, *h*_ab,10_ decreased by ~ 160, denoting a yellowish hue that is complimentary to the original hue. This *h*_ab,10_ value was retained at rGO loadings of > 3%.

#### Overall color changes

**Figure 7:**
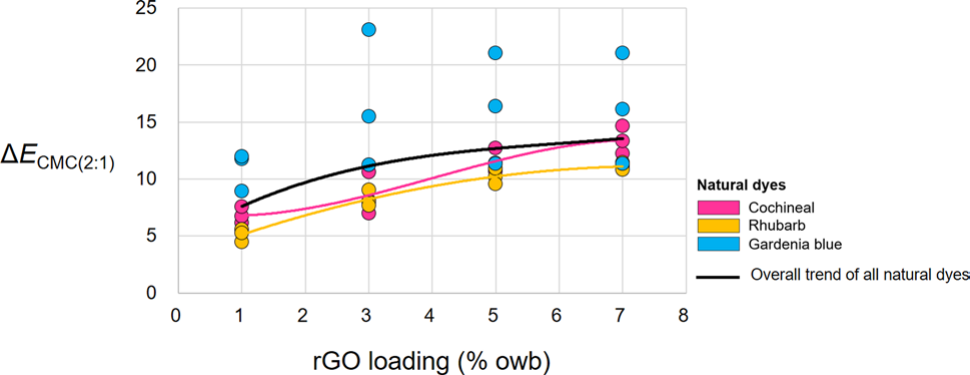
Effect of rGO loading on the Δ*E*_CMC(2:1)_ of the dyed fabrics. The solid lines show the best fit for the cochineal- and rhubarb-dyed fabrics, which exhibit significant effects, and the overall trend.

Figure 7 presents the effect of rGO loading on the Δ*E*_CMC(2:1)_ of the naturally dyed fabrics, revealing a significant effect for all fabrics (black line). In particular, Δ*E*_CMC(2:1)_ increased with increasing rGO loading. The average Δ*E*_CMC(2:1)_ of the cochineal-dyed fabrics (10.09 (S.D.: 2.89)) was larger than that of the rhubarb-dyed fabrics (8.66 (S.D.: 2.46)) by 1.43. Meanwhile, at rGO loadings of 1%, 3%, 5%, and 7%, the average Δ*E*_CMC(2:1)_ were 7.60 (S.D.: 2.75), 11.14 (S.D.: 5.19), 12.68 (S.D.: 3.72), and 13.56 (S.D.: 3.34), respectively, regardless of the type of natural dyes. The numerical values of Δ*E*_CMC(2:1)_ can guide the regulation of rGO loading in the production of multicolored graphene-based wearable e-textiles from the viewpoints of both functionality and aesthetics.

### Colorfastness of co-dyed fabrics

The colorfastness to laundering of the dyed fabrics was rated on a scale of 1 to 5 using AATCC Grayscale for Color Staining. Table 5 shows the results. The average rating of the dyed fabrics was 4.48, indicating excellent colorfastness to laundering. Also, regardless of the natural dye and rGO loading, all the dyed fabrics had a rating of 4 or higher, which is acceptable by ASTM for apparel. This demonstrates the feasibility of developing graphene-based e-textiles with various colors and excellent quality through dyeing with mixtures of natural dyes and rGO under different conditions.

**Table 5:** Colorfastness to laundering of the dyed fabrics.

Natural dye type	Colorfastness rating						Average
	Mordant	rGO loading (% owb)					
		0	1	3	5	7	
Cochineal	Al	4	4	4.5	4.5	4.5	4.43
	Cu	4	4	4.5	4.5	5	
	Fe	4.5	4.5	4.5	4.5	5	
Rhubarb	Al	4	4.5	4.5	4.5	4.5	4.47
	Cu	4	4	4.5	4.5	4.5	
	Fe	4	4.5	5	5	5	
Gardenia blue	Al	4	4	4.5	5	5	4.53
	Cu	4	4	4.5	4.5	5	
	Fe	4.5	4.5	4.5	5	5	
Average		4.11	4.22	4.56	4.67	4.83	4.48

The colorfastness was rated on a scale of 1 (lowest quality) to 5 (highest quality).

## Conclusion

In this study, cotton fabrics were dyed under various conditions using cochineal, rhubarb, and gardenia blue as the natural dyes and Al, Cu, and Fe salts as the mordants with different rGO loadings (0%, 1%, 3%, 5%, and 7% owb) to develop ecofriendly and colorful graphene-based wearable e-textiles. The physical color attributes of the 45 dyed samples were examined spectrophotometrically. The L^*^_10_ of the samples obtained without rGO increased in the order of cochineal < gardenia blue < rhubarb. Moreover, the average L^*^_10_ of the Fe-mordanted fabrics was lower by 4.91 than that of Al- and Cu-mordanted fabrics. Meanwhile, the C^*^_ab,10_ and *h*_ab,10_ of the co-dyed fabrics exhibited broad variations, with the widest ranges obtained for the rhubarb and gardenia blue natural dyes, respectively. Therefore, the dyeing conditions had significant impacts on the relevant color attributes. The C^*^_ab,10_ of the rhubarb-dyed fabrics significantly decreased with increasing rGO loading. Meanwhile, the initial greenish-blue color of the gardenia blue-dyed fabrics gradually turned yellowish-green and then yellow. Regardless of the natural dye used, rGO loading significantly affected Δ*E*_CMC(2:1)_; the values were 7.60, 11.14, 12.68, and 13.56 at rGO loadings of 1%, 3%, 5%, and 7%, respectively. Finally, the co-dyed fabrics were found to have excellent colorfastness to laundering with the average colorfastness rating of 4.48.

This study demonstrated the feasibility of developing graphene-based e-textiles with different color attributes through dyeing with mixtures of natural dyes and rGO under different conditions. The established ecofriendly approach facilitates the production of e-textiles with aesthetically appealing colors and useful functionality. Nonetheless, the color palette was restricted (particularly in terms of hue) by the limited number of natural dyes. Furthermore, this study focused on the physical color attributes of graphene-based e-textiles rather than their electrical performance. Future research will focus on the fabrication of e-textiles with a broader color palette using more diverse dyeing conditions (including natural dye type, rGO loading, and mordant type). Furthermore, the ideal dyeing conditions for the production of smart clothing will be determined by assessing the changes in the physical color attributes and electrical performance of the textiles.

## Data Availability

The authors confirm that data supporting the findings of this study are available within the article.
